# Lanthanide-Based Organic Salts: Synthesis, Characterization, and Cytotoxicity Studies

**DOI:** 10.3390/molecules28207152

**Published:** 2023-10-18

**Authors:** Andreia Forte, Sandra Gago, Celso Alves, Joana Silva, Joana Alves, Rui Pedrosa, César A. T. Laia, Isabel M. Marrucho, Luis C. Branco

**Affiliations:** 1 Associated Laboratory for Green Chemistry (LAQV) of the Network of Chemistry and Technology (REQUIMTE), Faculdade de Ciências e Tecnologia, Universidade Nova de Lisboa, Campus da Caparica, 2829-516 Caparica, Portugal; p110533@campus.fct.unl.pt (A.F.); s.gago@fct.unl.pt (S.G.); catl@fct.unl.pt (C.A.T.L.); 2 ITQB NOVA—Instituto de Tecnologia Química e Biológica António Xavier, Avenida da República, Estação Agronómica Nacional, 2780-157 Oeiras, Portugal; isabel.marrucho@tecnico.ulisboa.pt; 3 MARE—Marine and Environmental Sciences Centre/ARNET, ESTM, Politécnico de Leiria, Rua do Conhecimento, No. 4, 2520-614 Peniche, Portugal; celso.alves@ipleiria.pt (C.A.); joana.m.silva@ipleiria.pt (J.S.); fjtdgm@gmail.com (J.A.); rui.pedrosa@ipleiria.pt (R.P.); 4 Centro de Química Estrutural, Instituto Superior Técnico, Universidade de Lisboa, Avenida Rovisco Pais, 1049-001 Lisboa, Portugal

**Keywords:** magnetic organic salts, terbium salts, gadolinium salts, MRI contrast agents, cytotoxicity

## Abstract

The formulation of magnetic ionic liquids (MILs) or organic salts based on lanthanides as anions has been explored. In this work, a set of choline-family-based salts, and two other, different cation families, were combined with Gadolinium(III) and Terbium(III) anions. Synthetic methodologies were previously optimized, and all organic salts were obtained as solids with melting temperatures higher than 100 °C. The magnetic moments obtained for the Gd(III) salts were, as expected, smaller than those obtained for the Tb(III)-based compounds. The values for Gd(III) and Tb(III) magnetic salts are in the range of 6.55–7.30 MB and 8.22–9.34 MB, respectively. It is important to note a correlation between the magnetic moments obtained for lanthanides, and the structural features of the cation. The cytotoxicity of lanthanide-based salts was also evaluated using 3T3, 293T, Caco2, and HepG2 cells, and it was revealed that most of the prepared compounds are not toxic.

## 1. Introduction

In recent decades, ionic liquids (ILs) as low-melting organic salts have been studied and applied in a growing range of different areas [1]. Their negligible volatility, high chemical and thermal stability, and wide electrochemical range can be highlighted, but the significant advantage of these salts relies on the possibility of tuning their physicochemical and/or biological properties, by changing their formulation, i.e., through the suitable combination of specific organic cations and organic or inorganic anion units [2,3,4]. Currently, a new subclass of ILs, magnetic ionic liquids (MILs), have attracted greater attention. MILs are composed of at least one paramagnetic element in the cation or anion, and combine the properties of ILs and magnetic materials; in particular, the response to a strong magnetic field [5]. Since they were first reported in 2004 by Hayashi and Hamaguchi, MILs have been largely explored in different research areas, such as catalysis, separation processes and, more recently, in bio-applications [6,7,8,9]. Specifically, due to their magnetic and luminescence properties, metal complexes based on lanthanides have been explored for their use in the biomedical field—namely, to apply in imaging diagnosis techniques. MRI can provide images from the internal structures of the human body with a good spatial resolution, as well as an unlimited tissue penetration level. The image generated via this technique is based on the proton relaxation rates from water molecules, and the proton densities in different tissues [10]. Although, in some cases, a poor image contrast can be observed, the addition of some compounds (contrast agents) can improve this limitation, through a change in the nuclear relaxation rate of the water molecules [11]. MRI T_1_-shortening agents are mainly composed of a paramagnetic metal-chelate structure, with a particular focus on reducing the longitudinal relaxation time of the water molecules directly coordinated with, or in the vicinity of, the contrast agent. The T_1_ weighted image is one of the basic pulse sequences used in the MRI technique. This sequence allows the demonstration of the difference between the T_1_ relaxation times of tissues [12,13]. In the 3D image of the longitudinal relaxation time of the protons present in the tissues, it is possible to observe that short relaxation times appear bright, compared to longer relaxation times, where the image is darker. Sometimes, this contrast difference can be enough to perform an adequate diagnostic; however, in some cases, the addition of a contrast agent is required in order to enhance this contrast. This compound induces a reduction in the T_1_ relaxation time, a hypersignal is attained and, as a consequence, a brighter image can be observed [14,15]. Most commercially available MRI contrast agents are formulated with Gadolinium(III), Gd^3+^. This metal is the preferential choice for T_1_ contrast agents’ formulation, due to its seven unpaired electrons (the highest number for a metal ion), and its long electronic relaxation time. Owing to the combination of these properties, Gadolinium ions induce the largest effect compared to other paramagnetic ions, corresponding to the highest relaxation efficiency [10,12,16]. In general, the Lanthanides (Ln), or rare-earth metals, are characterized by the progressive filling of their 4f valence orbitals, and by being highly paramagnetic, due to their unpaired electrons. Particularly, in the case of Gd(III), a higher magnetic moment, magnetic susceptibilities, and electronic relaxation times are observed [17,18,19,20,21,22,23,24]. The use of this metal in the formulation of contrast agents for medical applications has a major drawback in the form of Gd(III) toxicity, due to several mechanisms of action, including calcium channel inhibition [12].

In this context, it is important to evaluate the toxicity of alternative materials, via the selection of appropriate cell lines. The use of cancer cells (e.g., HepG2 and CaCo-2) and non-cancer cells (e.g., 293T and 3T3 cells) as cellular models is important in evaluating the toxicity of distinct compounds. In general, the HepG2 cell line is currently one of the most frequently used human cell models in the hepatotoxicity screening of drugs [25]. Caco-2 cells are derived from human colorectal adenocarcinoma, and can closely mimic human intestinal epithelial cells, providing a powerful tool for evaluating risk in cytotoxic compounds [26]. The human embryonic kidney 293T cell is a cell line derived from the human embryonic kidney cell, and is also widely used for the evaluation of the cytotoxic effects of molecules [27], while 3T3 cells are derived from mouse embryonic fibroblasts. The relevance of these cells as cellular models for cytotoxicity assays is also strongly supported by the “Test Guideline No. 432 In Vitro 3T3 NRU Phototoxicity Test”, approved by the Organization for Economic Cooperation and Development (OECD GD 129, 2010), in which 3T3 cells are used as a cellular model to evaluate the phototoxicity of chemicals. Moreover, this guideline highlights that “The in vitro 3T3 NRU phototoxicity test was shown to be predictive of acute phototoxicity effects in animals and humans in vivo”, supporting the belief that this cellular model is viable for predicting the cytotoxicity of compounds for application in humans.

**Figure 1:**
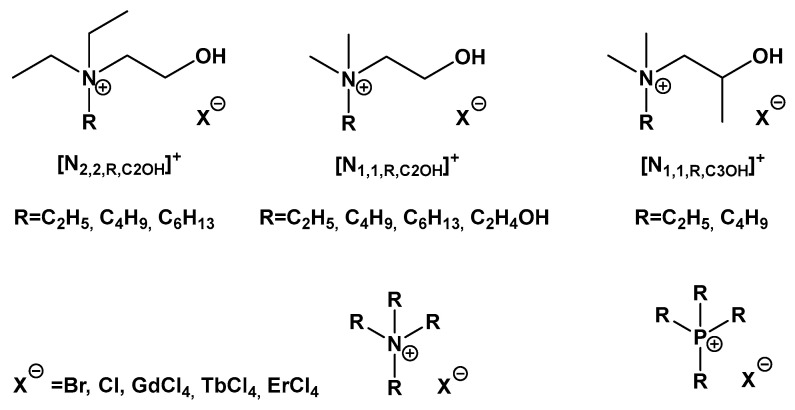
The structures of the biocompatible organic salts synthesized in this work.

Herein, a series of new magnetic organic salts based on different choline-derivative cations combined with Gadolinium(III) and Terbium(III) anions were prepared and characterized. Additionally, examples of ammonium and phosphonium cations were used for comparison. Figure 1 illustrates the chemical structures of the prepared biocompatible magnetic organic salts.

## 2. Results and Discussion

### 2.1. Synthesis and Characterization of Magnetic Salts

A set of choline-derivative cations described in a previous work [28] were used to synthesize new magnetic salts based on two different lanthanides, Gadolinium(III) and Terbium(III). Due to their similar electronic configuration, the Lanthanide series metals show identical physical–chemical properties. Most of the metals from this family are paramagnetic elements, due to their unpaired electrons. This feature, and their 4f electron configuration, make them good candidates for use in metal complex formulations. Over the years, the main choice for application in MRI contrast agent design has been focused on trivalent cation Gd^3+^. This metal presents several advantages, such as a high number of unpaired electrons (seven electrons, the highest possible in the lanthanide family), the high associated magnetic moment, and the predisposition to form metal complexes with a high stability [29]. Terbium(III) arises as an alternative, as it possesses a higher magnetic moment than Gadolinium(III), and it is also a strongly paramagnetic ion [30].

**Scheme 1:**
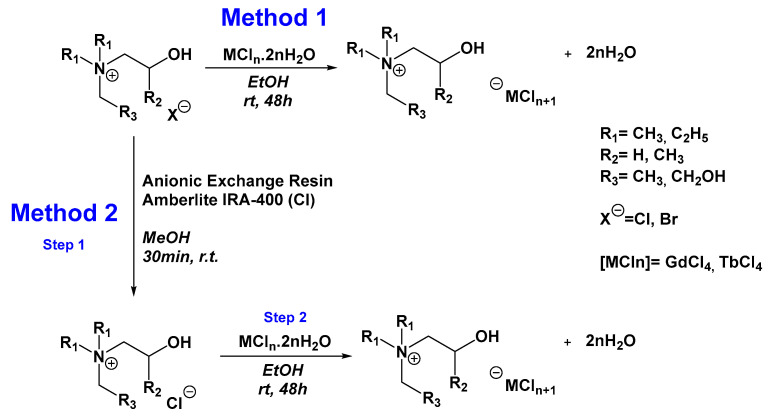
The synthetic methodology adopted to develop lanthanide-based magnetic salts.

The synthetic methodology used in this work, in order to develop these novel lanthanide-based magnetic organic salts, is illustrated in Scheme 1. 

Beyond choline-derivative cations, other organic cations, such as tetra-alkyl ammonium ([N_2,2,2,2_]), and two phosphonium cations ([P_OH,OH,OH,OH_] and [P_4,4,4,4_]), were also selected. As already described, two synthetic methods were applied to obtain the metal-based organic salts: for the cases where the organic cation had a chloride as a counter-ion, only the metal complexation reaction was performed, but when this anion was bromide, a previous step was required, to convert these salts into the correspondent chloride ones. This synthetic step was accomplished using an ionic exchange resin. It is important to emphasize that the complexation reactions were performed in a single-step procedure, with no additional purification steps. The different families of magnetic salts were characterized according to their physical and thermal properties. The results are presented in Table 1. All compounds were obtained as solids, and most of them had transition temperatures (melting or decomposition) higher than 100 °C. Due to their cation scaffold, most of the synthesized magnetic salts reveal a high hygroscopic nature. Choline-derivative-based salts are highly soluble in water and alcohols, but present a low solubility in apolar organic solvents. 

One of the bases of lanthanide chemistry and applications is related to their luminescence. This important property arises from forbidden 4f–4f electronic transitions, and results in narrow line widths associated with long lifetimes. These properties makes these elements attractive for several optical applications [31]. It is expected that Gd(III) and Tb(III) complexes offer similar chemical features, as well as biodistribution profiles [32]. However, visual observation apparently suggests that Tb(III)-based compounds present a more intense luminescent behaviour than Gadolinium(III) salts. This could be useful in further bio-applications. Visual observation of these compounds seems to indicate a green light emission that can be explored in the future. In Table 2 are some images from the prepared Tb(III)-based organic salts under (A) white light, and (B) UV light (366 nm).

### 2.2. Magnetic Moment

As magnetic properties comprise one of the major important features of contrast agents, the magnetic susceptibility of the prepared lanthanide-based magnetic organic salts was measured. The effective magnetic moment (µ_eff_) of these compounds was also determined. All results are summarized in Table 3. It is important to note that, as expected, the µ_eff_ values found for Gd(III) salts are smaller than for the correspondent Tb(III) compounds. The µ_eff_ values obtained for Gd(III) magnetic salts are in the range of 6.55–7.30 MB. [N_1,1,1,C2OH_][GdCl_4_], [N_1,1,2,C3OH_][GdCl_4_], and [N_1,1,4,C3OH_][GdCl_4_] salts showed the highest magnetic moments in the case of the Gd(III) series. Regarding Tb(III)-based salts, the values obtained are in the range of 8.22–9.34 MB. In general, the values are comparatively lower than those corresponding to the free metal. This observation can be justified via metal–ligand coordination, as well as the possible interaction with the organic cation. Even so, Tb(III)-based salts presented very high µ_eff_ values and, also, these are higher than those obtained for Gd(III). Thus, although their magnetic properties can be affected by the coordination and salt formulation, Tb(III) seems to maintain higher magnetic moment values. More recently, the interest in these metal-based compounds has increased significantly; however, there are still few studies using Tb complexes in magnetic applications.

**Figure 2:**
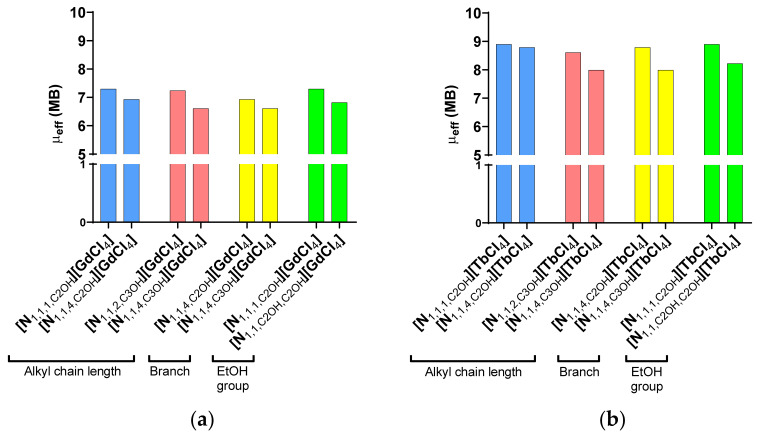
The variation in μ_eff_ with the cation structural properties for (**a**) Gd(III)-, and (**b**) Tb(III)-based magnetic organic salts.

Regarding choline-derivative salts, the results show a standard behaviour in the μ_eff_ values that depended on the cation structural features. The correlation between the organic cation scaffold and the values obtained seems to be the same for both metals (Figure 2). Thus, when the side alkyl chain increases, the µ_eff_ decreases. The same trend is observed when a branch is introduced into the cation structure. Finally, when an additional ethanol group is introduced, the µ_eff_ decreases. This behaviour was already found for another two metals, Fe(III) and Mn(II).

### 2.3. Cytotoxicity of Lanthanide-Based Salts

**Figure 3:**
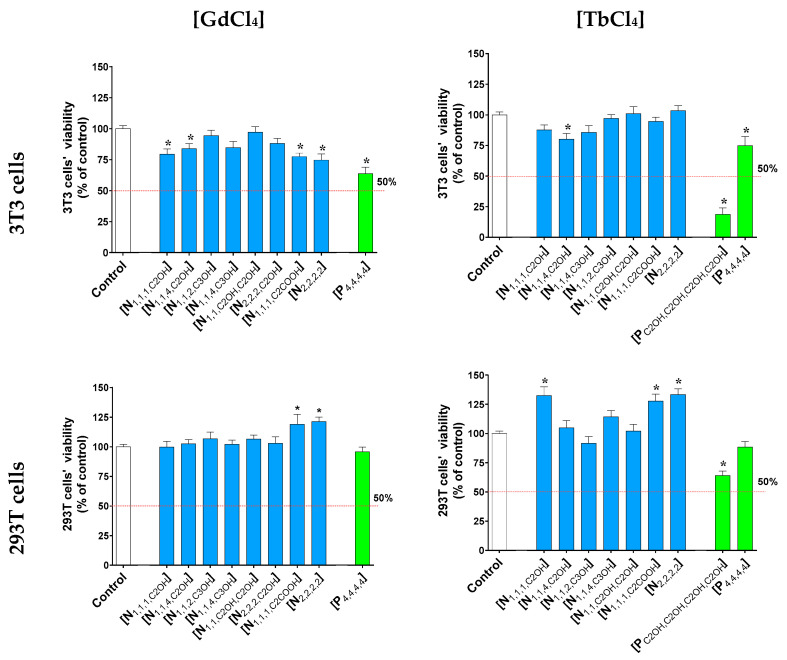
The cytotoxicity of Gd(III)- and Tb(III)-based organic salts (100 µg/mL) on 3T3 and 293T cells’ viability after treatment for 24 h. Values represent mean ± standard error of the mean (SEM) of at least three independent experiments carried out in triplicate. Symbols represent significant differences (ANOVA, Dunnett’s test, *p* < 0.05) compared to the control (*).

**Figure 4:**
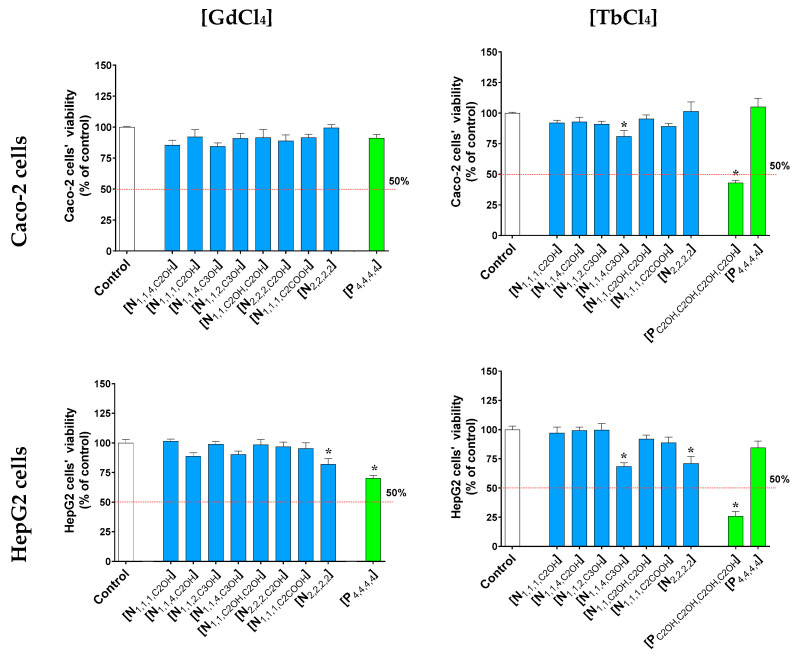
The cytotoxicity of Gd(III)- and Tb(III)-based organic salts (100 µg/mL) on HepG2 and Caco-2 cells’ viability after treatment for 24 h. Values represent mean ± standard error of the mean (SEM) of at least three independent experiments carried out in triplicate. Symbols represent significant differences (ANOVA, Dunnett’s test, *p* < 0.05) compared to the control (*).

The cytotoxicity of lanthanide-based prepared salts (100 µg/mL) was evaluated based on 3T3, 293T, Caco-2, and HepG2 cells after 24 h treatment. The effects of the synthesized organic salts on the studied cells’ viability were estimated via an MTT assay, and the results are displayed in Figure 3 and Figure 4, according to the cells’ nature (normal or carcinogenic). From the obtained results, it can be emphasized that most of the tested compounds did not induce cytotoxicity in 3T3, 293T, Caco2, or HepG2 cells. Even so, some exceptions can be highlighted: Gd(III)-based compounds induced a more marked effect on the 3T3 cells’ viability than that of the remaining cell lines. [N_1,1,1,C2OH_][GdCl_4_], [N_1,1,4,C2OH_][GdCl_4_], [N_1,1,1,C2COOH_][GdCl_4_], [N_2,2,2,2_][GdCl_4_], and [P_4,4,4,4_][GdCl_4_] salts significantly reduced the cells’ viability in a range from 16.17 to 32.28%, compared with the control. For the same cellular model, the Tb(III)-based salts [N_1,1,4,C2OH_][TbCl_4_], [P_OH,OH,OH,OH_][TbCl_4_], and [P_4,4,4,4_][TbCl_4_] also reduced the 3T3 cells’ viability by 19.77%, 81.22%, and 25.08%, respectively. For the 293T cells, a different behaviour could be observed: out of all of the synthesized magnetic compounds, [N_1,1,1,C2OH_][TbCl_4_], [N_2,2,2,2_][GdCl_4_], [N_2,2,2,2_][TbCl_4_], [N_1,1,1,C2COOCH3_][GdCl_4_], and [N_1,1,1,C2COOCH3_][TbCl_4_] presented a percentage of cell viability higher than 100%, compared with the control situation. Thus, these compounds seem to increase mitochondrial activity.

[P_OH,OH,OH,OH_][TbCl_4_] was the only example that exhibited a cytotoxic effect in 293T cells (63.95% of viable cells) compared to the control situation. Regarding tumoral cells, the prepared compounds revealed a smaller cytotoxic effect, compared to that shown on normal cells. For Caco-2 cells, only [N_1,1,4,C3OH_][TbCl_4_] and [P_OH,OH,OH,OH_][TbCl_4_] induced a significant reduction in viability, of 19.06% and 57.10%, respectively, compared to the control situation. Finally, the [N_2,2,2,2_][GdCl_4_], [P_4,4,4,4_][GdCl_4_], [N_1,1,4,C3OH_][TbCl_4_], [N_2,2,2,2_][TbCl_4_], and [P_OH,OH,OH,OH_][TbCl_4_] salts significantly reduced the viability of HepG2 cells compared to the control situation. In this case, a cell viability range from 25.80 to 81.96% was observed.

## 3. Materials and Methods

### 3.1. General Remarks

All the commercial organic solvents were used as supplied from Sigma-Aldrich at an analytical purity grade. Commercially available reagents were purchased from different chemical companies, and were then used as received. Fourier transform infrared spectroscopy (FTIR) spectra (in Supplementary Materials) were carried out via a Bruker Tensor 27. Elemental analysis (C, H, N analyzer) of each synthesized organic salt was performed at Laboratório de Análises at LAQV-REQUIMTE. For the salts which were solid at room temperature, the melting point determination was performed using a Stuart Scientific Melting Point SMP1.

### 3.2. Synthesis of Metal-Complex Salts

General method: Choline-chloride-derivative salt (as indicated in Figure 1) and ethanol were added to a round-bottomed flask. After complete dissolution, the selected hydrated lanthanide salt (GdCl_3_ or TbCl_3_) was added, under vigorous magnetic stirring at room temperature. The reaction mixture was stirred for 48 h, and then the solvent was evaporated, and the product was dried under a vacuum.

#### 3.2.1. Gadolinium(III)-Based Organic Salts

**[N_1,1,1,C2OH_][GdCl_4_]:**

***N*** **-hydroxyethyl-*N*,*N*,*N*-trimethylammonium tetrachlorogadolinate(III)**

[N_1,1,1,C2OH_][Cl] (0.21 g, 1.50 mmol); GdCl_3_.6H_2_O (0.55 g, 1.49 mmol); yield: quantitative; white solid; FTIR (KBr), ῡ = 3355, 1627, 1476, 1280, 1200, 1132, 1081, 955 cm^−1^. Elemental analysis calcd (%) for C_5_H_14_Cl_4_GdNO.2.8H_2_O (453.50 g·mol^−1^): C 13.24, N 3.09, H 4.32; found: C 12.79, N 2.92, H 4.14.

**[N_1,1,4,C2OH_][GdCl_4_]:**

***N*** **-butyl-*N*-hydroxyethyl-*N*,*N*-dimethylammonium tetrachlorogadolinate(III)**

[N_1,1,4,C2OH_][Cl] (0.20 g, 1.35 mmol); GdCl_3_.6H_2_O (0.50 g, 1.36 mmol); yield: quantitative; white solid; FTIR (KBr), ῡ = 3363, 2964, 1627, 1468, 1084, 975, 921 cm^−1^. Elemental analysis calcd (%) for C_8_H_20_Cl_4_GdNO.0.5H_2_O (454.35 g·mol^−1^): C 21.15, N 3.08, H 4.67; found: C 21.32, N 3.02, H 5.50.

**[N_1,1,4,C3OH_][GdCl_4_]:**

***N*** **-butyl-*N*-hydroxyethyl-*N*,*N*-dimethylammonium tetrachlorogadolinate(III)**

[N_1,1,4,C3OH_][Cl] (0.26 g, 1.32 mmol); GdCl_3_.6H_2_O (0.49 g, 1.32 mmol); yield: quantitative; yellow solid; FTIR (KBr), ῡ = 3375, 2967, 1635, 1508, 1148, 1071, 993, 890 cm^−1^. Elemental analysis calcd (%) for C_9_H_22_Cl_4_GdNO.0.5H_2_O (468.38 g·mol^−1^): C 23.08, N 2.99, H 4.96; found: C 22.59, N 3.07, H 4.90.

**[N_1,1,2,C3OH_][GdCl_4_]:**

***N*** **-ethyl-*N*-hydroxypropyl-*N*,*N*-dimethylammonium tetrachlorogadolinate(III)**

[N_1,1,2,C2OH_][Cl] (0.17 g, 1.04 mmol); GdCl_3_.6H_2_O (0.38 g, 1.03 mmol); yield: quantitative; yellow solid; FTIR (KBr), ῡ = 3334, 1635, 1476, 1410, 1144, 1083, 1023, 977 cm^−1^. Elemental analysis calcd (%) for C_7_H_18_Cl_4_GdNO.3.5H_2_O (494.38 g·mol^−1^): C 17.01, N 2.83, H 5.11; found: C 16.67, N 2.67, H 4.42.

**[N_1,1,C2OH,C2OH_][GdCl_4_]:**

***N*** **,*N*-dihydroxyethyl-*N*,*N*-dimethylammonium tetrachlorogadolinate(III)**

[N_1,1,C2OH,C2OH_][Cl] (0.25 g, 1.48 mmol); GdCl_3_.6H_2_O (0.55 g, 1.47 mmol); yield: quantitative; white solid; FTIR (KBr), ῡ = 3357, 1631, 1477, 1078, 957 cm^−1^. Elemental analysis calcd (%) for C_6_H_16_Cl_4_GdNO_2_.0.5H_2_O (442.29 g·mol^−1^): C 16.29, N 3.17, H 3.88; found: C 16.30, N 2.80, H 4.33.

**[N_2,2,2,C2OH_][GdCl_4_]:**

***N*** **,*N*,*N*-triethyl-*N*-hydroxyethylammonium tetrachlorogadolinate(III)**

[N_2,2,2,C2OH_][Cl] (0.21 g, 1.14 mmol); GdCl_3_.6H_2_O (0.42 g, 1.13 mmol); yield: quantitative; white solid; FTIR (KBr), ῡ = 3471, 1635, 1477, 1402, 1156, 1081, 924 cm^−1^. Elemental analysis calcd (%) for C_8_H_20_Cl_4_GdNO.3.5H_2_O (508.41 g·mol^−1^): C 18.90, N 2.76, H 5.36; found: C 18.76, N 2.30, H 4.64.

**[N_1,1,1,C2COOCH3_][GdCl_4_]:**

**(2-Acetoxyethyl)-*N*,*N*,*N*-trimethylammonium tetrachlorogadolinate(III)**

[N_1,1,1,C2COOCH3_][Cl] (0.26 g, 1.46 mmol); GdCl_3_.6H_2_O (0.54 g, 1.46 mmol); yield: quantitative; white solid; FTIR (KBr), ῡ = 3384, 1721, 1637, 1478, 1401, 1254, 1051, 954, 669 cm^−1^. Elemental analysis calcd (%) for C_7_H_16_Cl_4_GdNO.2.5H_2_O (490.34 g·mol^−1^): C 17.15, N 2.86, H 4.33; found: C 16.95, N 2.76, H 4.8.

**[N_2,2,2,2_][GdCl_4_]:**

**Tetraethylammonium tetrachlorogadolinate(III)**

[N_2,2,2,2_][Cl] (0.26 g, 1.41 mmol); GdCl_3_.6H_2_O (0.53 g, 1.42 mmol); yield: quantitative; white solid; FTIR (KBr), ῡ = 3379, 1628, 1488, 1142, 1396, 1175, 1057, 1002, 787, 635 cm^−1^. Elemental analysis calcd (%) for C_8_H_20_Cl_4_GdN.0.25H_2_O (433.85 g·mol^−1^): C 21.15, N 3.23, H 4.77; found: C 21.66, N 3.07, H 5.14.

**[P_4,4,4,4_][GdCl_4_]:**

**Tetrabutylphosphonium tetrachlorogadolinate(III)**

[P_4,4,4,4_][Cl] (0.25 g, 0.85 mmol); GdCl_3_.6H_2_O (0.32 g, 0.85 mmol); yield: quantitative; yellow solid; FTIR (KBr), ῡ = 3375, 2960–2873, 1625, 1464, 1232, 1095, 1003, 968, 908 cm^−1^. Elemental analysis calcd (%) for C_16_H_36_Cl_4_GdP.4.5H_2_O (639.63 g·mol^−1^): C 30.04, H 7.11; found: C 30.34, H 7.75.

#### 3.2.2. Terbium(III)-Based Organic Salt

**[N_1,1,1,C2OH_][TbCl_4_]:**

***N*** **-hydroxyethyl-*N*,*N*,*N*-trimethylammonium tetrachloroterbate(III)**

[N_1,1,1,C2OH_][Cl] (0.21 g, 1.50 mmol); TbCl_3_.6H_2_O (0.57 g, 1.50 mmol); yield: quantitative; white solid; FTIR (KBr), ῡ = 3380, 1628, 1476, 1081, 1047, 955 cm^−1^. Elemental analysis calcd (%) for C_5_H_14_Cl_4_NOTb.2.3H_2_O (446.38 g·mol^−1^): C 13.45, N 3.14, H 4.21; found: C 13.14, N 2.99, H 4.67.

**[N_1,1,4,C2OH_][TbCl_4_]:**

***N-*** **butyl-*N*-hydroxyethyl-*N*,*N*-dimethylammonium tetrachloroterbate(III)**

[N_1,1,4,C2OH_][Cl] (0.30 g, 2.06 mmol); TbCl_3_.6H_2_O (0.77 g, 2.07 mmol); yield: quantitative; white solid; FTIR (KBr), ῡ = 3370, 2965–2876, 1633, 1486, 1131, 1084, 1051, 976, 920 cm^−1^. Elemental analysis calcd (%) for C_8_H_20_Cl_4_NOTb.2.5H_2_O (492.07 g·mol^−1^): C 19.53, N 2.85, H 5.13; found: C 19.53, N 2.74, H 5.03.

**[N_1,1,4,C3OH_][TbCl_4_]:**

***N-*** **butyl-*N*-hydroxyethyl-*N*,*N*-dimethylammonium tetrachloroterbate(III)**

[N_1,1,4,C3OH_][Cl] (0.26 g, 1.32 mmol); TbCl_3_.6H_2_O (0.49 g, 1.31 mmol); yield: quantitative; yellow solid; FTIR (KBr), ῡ = 3346, 2967–2877, 1628, 1485, 1288, 1148, 1072, 993, 891 cm^−1^. Elemental analysis calcd (%) for C_9_H_22_Cl_4_NOTb.2H_2_O (497.09 g·mol^−1^): C 21.74, N 2.82, H 5.28; found: C 22.15, N 2.85, H 6.00.

**[N_1,1,2,C3OH_][TbCl_4_]:**

***N*** **-ethyl-*N*-hydroxypropyl-*N*,*N*-dimethylammonium tetrachloroterbate(III)**

[N_1,1,2,C2OH_][Cl] (0.25 g, 1.49 mmol); TbCl_3_.6H_2_O (0.56 g, 1.49 mmol); yield: quantitative; yellow solid; FTIR (KBr), ῡ = 3357, 1633, 1485, 1148, 1114, 1088, 1020, 980 cm^−1^. Elemental analysis calcd (%) for C_7_H_18_Cl_4_NOTb.4H_2_O (505.07 g·mol^−1^): C 16.65, N 2.77, H 5.20; found: C 16.60, N 2.70, H 4.43.

**[N_1,1,C2OH,C2OH][_TbCl_4_]:**

***N*** **,*N*-dihydroxyethyl-*N*,*N*-dimethylammonium tetrachloroterbate(III)**

[N_1,1,C2OH,C2OH_][Cl] (0.25 g, 1.48 mmol); TbCl_3_.6H_2_O (0.55 g, 1.47 mmol); yield: quantitative; white solid; FTIR (KBr), ῡ = 3371, 1639, 1479, 1076, 959 cm^−1^. Elemental analysis calcd (%) for C_6_H_16_Cl_4_NO_2_Tb (434.96 g·mol^−1^): C 16.57, N 3.22, H 3.72; found: C 16.25, N 3.04, H 3.64.

**[N_1,1,1,C2COOCH3_][TbCl_4_]:**

**(2-Acetoxyethyl)-*N*,*N*,*N*-trimethylammonium tetrachloroterbate(III)**

[N_1,1,1,C2COOCH3_][Cl] (0.25 g, 1.39 mmol); TbCl_3_.6H_2_O (0.54 g, 1.45 mmol); yield: quantitative; white solid; FTIR (KBr), ῡ = 3396, 1636, 1478, 1401, 1255, 1133, 1083, 1052, 954, 643 cm^−1^. Elemental analysis calcd (%) for C_7_H_16_Cl_4_NOTb.2.5H_2_O (492.02 g·mol^−1^): C 17.09, N 2.85, H 4.31; found: C 17.16, N 3.39, H 4.54.

**[N_2,2,2,2][_TbCl_4_]:**

**Tetraethylammonium tetrachloroterbate(III)**

[N_2,2,2,2_][Cl] (0.26 g, 1.41 mmol); TbCl_3_.6H_2_O (0.53 g, 1.42 mmol); yield: quantitative; white solid; FTIR (KBr), ῡ = 3384, 1636, 1486, 1399, 1175, 1002, 786, 670 cm^−1^. Elemental analysis calcd (%) for C_8_H_20_Cl_4_NTb.H_2_O (449.04 g·mol^−1^): C 21.40, N 3.12, H 4.95; found: C 21.04, N 2.94, H 4.77.

**[P_OH,OH,OH,OH_][TbCl_4_]:**

**Tetrahydroxymethylphosphonium tetrachloroterbate(III)**

[P_OH,OH,OH,OH_][Cl] (0.25 g, 1.31 mmol); TbCl_3_.6H_2_O (0.50 g, 1.31 mmol); yield: quantitative; white solid; FTIR (KBr), ῡ = 3384, 1637, 1400, 1048, 670 cm^−1^. Elemental analysis calcd (%) for C_4_H_12_Cl_4_O_4_PTb.4.5H_2_O (536.95 g·mol^−1^): C 8.95, H 3.95; found: C 9.31, H 4.34.

**[P_4,4,4,4_][TbCl_4_]:**

**Tetrabutylphosphonium tetrachloroterbate(III)**

[P_4,4,4,4_][Cl] (0.25 g, 0.86 mmol); TbCl_3_.6H_2_O (0.33 g, 0.87 mmol); yield: quantitative; yellow solid; FTIR (KBr), ῡ = 3355, 2961–2872, 1629, 1465, 1408, 1232, 1095, 967, 904 cm^−1^. Elemental analysis calcd (%) for C_16_H_36_Cl_4_PTb.4H_2_O (632.30 g·mol^−1^): C 30.39, H 7.03; found: C 31.15, H 7.58.

### 3.3. Magnetic Moment Determination

The mass susceptibility χ***_g_*** of the compounds was determined using a magnetic susceptibility balance at room temperature (20 °C), from Sherwood Scientific. The effective magnetic moment (**μ_eff_**) values were obtained using the following Equations (1)–(3).

(1) $$\chi_{g}=\frac{C.l.(R-R_{0})}{10^{9}\cdot m}$$

where ***C*** is the constant of calibration balance (=1.14), ***l*** is the length of the sample (cm), ***R*** is the reading for the tube with the sample, ***R*_0_** is the empty tube reading, and m is the mass of the sample (***g***).

(2) $$\chi_{m}=\chi_{g}.M_{w}$$

where χ**_m_** is the molar susceptibility, and **M_w_** is the molecular weight of the compound.

(3) $${µ}_{eff}=2.828\sqrt{\chi_{m}.T}$$

where **µ_eff_** is the effective magnetic moment, and **T** is the measurement temperature (in Kelvin, **T** (°C) + 273).

### 3.4. Cytotoxicity

The 3T3, Caco-2, HepG2, and 293T cell lines we used were previously acquired from the DSMZ biobank. The cells were cultured according to the supplier’s instructions. The 3T3 cells were cultured in Dulbecco’s Modified Eagle’s Medium: Nutrient Mix F-12 (DMEM/F-12) (Merck, Darmstadt, Germany), the Caco-2 cells were cultivated in Minimum Essential Medium (MEM) (Merck, Darmstadt, Germany), the HepG2 cells were grown in RPMI medium (Sigma-Aldrich, Saint Louis, MO, USA), and the 293T cells were cultivated in Dulbecco´s Modified Eagle´s Medium (Merck, Darmstadt, Germany). All media were supplemented with 10% bovine fetal serum (Hyclone, Nelson, UK), 100 IU/mL penicillin, and 100 μg/mL streptomycin (Merck, Darmstadt, Germany). 

For the subcultures, 3T3 cells, Caco-2 cells, HepG2 cells, and 293T cells were dissociated by means of trypsin–EDTA treatment (Sigma, Saint Louis, MO, USA), split in a 1:5, 1:3, 1:3, 1:6 ratio, respectively, and seeded into Petri dishes with a 25 cm^2^ growth area. The cells were maintained in a humidified atmosphere with 5% CO_2_, at 37 °C. 

The cytotoxicity of the lanthanide-based prepared salts was evaluated based on 3T3, Caco-2, HepG2, and 293T cells’ viability after the cells reached total confluence on 96-well plates. Cells were treated with salts at 100 μg/mL for 24 h. The effects were estimated by means of an MTT (Sigma, Darmstadt, Germany) colorimetric assay based on the conversion of tetrazolium salts to blue formazan products by active mitochondria. The results were expressed as the percentage of the control (%).

Data and statistical analysis: the results are presented as mean ± standard error of the mean (SEM). At least three independent experiments were carried out in triplicate. Statistical analysis was performed using one-way analysis of variance (ANOVA) with Dunnett’s multiple comparison of group means, to determine significant differences relative to the control treatment. Differences were considered statistically significant at a level of 0.05 (*p*-value < 0.05). Calculations were performed using GraphPad v5.1 (GraphPad Software, La Jolla, CA, USA) software.

## 4. Conclusions

A set of Gd(III)- and Tb(III)-based magnetic organic salts was prepared and characterized. Choline derivatives and two other organic cations were tested in metal complexation reactions, in order to obtain new, and more biocompatible, magnetic organic salts for potential application as contrast agents in MRI. Gd(III) is the most common metal used in contrast agent formulation, but the high metal-free associated toxicity is reported as a significant limitation. Tb(III) complexes arise at this point as a good alternative to Gd(III) complexes, due to the nature and magnetic properties of the metal. For choline-derivative magnetic salts, a standard correlation between the µ_eff_ of compounds, and their organic cation structure is observed. The cytotoxic effects of the prepared compounds in four different cell lines were evaluated, and no significant toxicity was observed for the majority of the prepared compounds in the studied cells. 

## Figures and Tables

**Table 1:** The physical properties of lanthanide-based organic salts.

	Compound	Physical State	Tm [Td] ^(a)^ (°C)
	[N_1,1,1,C2OH_][GdCl_4_]	White solid	145
	[N_1,1,4,C2OH_][GdCl_4_]	White solid	118
	[N_1,1,C2OH,C2OH_][GdCl_4_]	White solid	Hygroscopic
	[N_1,1,2,C3OH_][GdCl_4_]	Pale yellow solid	147
Gd(III)	[N_1,1,4,C3OH_][GdCl_4_]	Yellow solid	148
	[N_2,2,2,C2OH_][GdCl_4_]	White solid	95
	[N_1,1,1,C2COOCH3_][GdCl_4_]	White solid	152
	[N_2,2,2,2_][GdCl_4_]	White solid	114
	[P_4,4,4,4_][GdCl_4_]	Yellow solid	Hygroscopic
	[N_1,1,1,C2OH_][TbCl_4_]	White solid	114
	[N_1,1,4,C2OH_][TbCl_4_]	White solid	115
	[N_1,1,C2OH,C2OH_][TbCl_4_]	White solid	Hygroscopic
	[N_1,1,2,C3OH_][TbCl_4_]	Pale yellow solid	139
Tb(III)	[N_1,1,4,C3OH_][TbCl_4_]	Yellow solid	155
	[P_OH,OH,OH,OH_][TbCl_4_]	White solid	146
	[N_1,1,1,C2COOCH3_][TbCl_4_]	White solid	142
	[N_2,2,2,2_][TbCl_4_]	White solid	110
	[P_4,4,4,4_][TbCl_4_]	Yellow solid	Hygroscopic

**^a^** transition temperatures (melting or decomposition).

**Table 2:** Images from the prepared Tb(III)-based organic salts under (A) white light, and (B) UV light (366 nm).

Compound	[N1,1,1,C2OH]	[N1,1,4,C2OH]	[N1,1,C2OH,C2OH]	[N1,1,2,C3OH]	[N1,1,4,C3OH]	[POH,OH,OH,OH]	[P4,4,4,4]
(A)	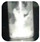	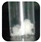	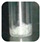	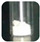	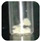	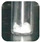	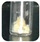
(B)	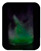	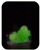	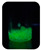	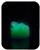	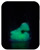	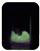	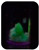

**Table 3:** The magnetic susceptibility and effective magnetic moments of magnetic organic salts based on Gd(III) and Tb(III).

	Compound	10^−6^ × χ_g_ (c.g.s) ^(a)^	µ_eff_ (MB) ^(b)^
Gd(III)	Gd(III) free ion	-	7.94 [33]
	[N_1,1,1,C2OH_] [GdCl_4_]	55.78	7.30
	[N_1,1,4,C2OH_] [GdCl_4_]	45.48	6.93
	[N_1,1,C2OH,C2OH_] [GdCl_4_]	45.25	6.82
	[N_1,1,2,C3OH_] [GdCl_4_]	51.24	7.24
	[N_1,1,4,C3OH_] [GdCl_4_]	47.83	7.22
	[N_2,2,2,C2OH_] [GdCl_4_]	40.61	6.55
	[N_1,1,1,C2COOCH3_][GdCl_4_]	n.d.	n.d.
	[N_2,2,2,2_] [GdCl_4_]	n.d.	n.d.
	[P_4,4,4,4_] [GdCl_4_]	37.45	7.04
Tb(III)	Tb(III) free ion	-	9.72 [34]
	[N_1,1,1,C2OH_] [TbCl_4_]	82.41	8.90
	[N_1,1,4,C2OH_] [TbCl_4_]	82.23	9.34
	[N_1,1,C2OH,C2OH_] [TbCl_4_]	65.59	8.22
	[N_1,1,2,C3OH_] [TbCl_4_]	72.21	8.61
	[N_1,1,4,C3OH_] [TbCl_4_]	58.45	7.99
	[P_OH,OH,OH,OH_] [TbCl_4_]	59.29	8.01
	[N_1,1,1,C2COOCH3_][TbCl_4_]	n.d.	n.d.
	[N_2,2,2,2_] [TbCl_4_]	n.d.	n.d.
	[P_4,4,4,4_] [TbCl_4_]	56.50	8.66

^(a)^ The magnetic susceptibility (χ_g_) measured via a Sherwood magnetic susceptibility balance. ^(b)^ Effective magnetic moment (µ_eff_) obtained using the magnetic susceptibility and the equation µ_eff_ = 2.828$\sqrt{XgT}$. n.d., not determined.

## Data Availability

Data are available in a publicly accessible repository.

## Supplementary Materials

The following supporting information can be downloaded at: https://www.mdpi.com/article/10.3390/molecules28207152/s1, Figure S1: FT-IR spectra of [N_1,1,1,C2OH_][GdCl_4_]; Figure S2: FT-IR spectra of [N_1,1,4,C2OH_][GdCl_4_]; Figure S3: FT-IR spectra of [N_1,1,C2OH,C2OH_][GdCl_4_]; Figure S4: FT-IR spectra of [N_1,1,2,C3OH_][GdCl_4_]; Figure S5: FT-IR spectra of [N_1,1,4,C3OH_][GdCl_4_]; Figure S6: FT-IR spectra of [N_2,2,2,C2OH_][GdCl_4_]; Figure S7: FT-IR spectra of [N_1,1,1,C2COOCH3_][GdCl_4_]; Figure S8: FT-IR spectra of [N_2,2,2,2_][GdCl_4_]; Figure S9: FT-IR spectra of [P_4,4,4,4_][GdCl_4_]; Figure S10: FT-IR spectra of [N_1,1,1,C2OH_][TbCl_4_]; Figure S11: FT-IR spectra of [N_1,1,4,C2OH_][TbCl_4_]; Figure S12: FT-IR spectra of [N_1,1,2,C3OH_][TbCl_4_]; Figure S13: FT-IR spectra of [N_1,1,4,C3OH_][TbCl_4_]; Figure S14: FT-IR spectra of [P_C2OH,C2OH,C2OH,C2OH_][TbCl_4_]; Figure S15: FT-IR spectra of [N_1,1,1,C2COOCH3_][TbCl_4_]; Figure S16: FT-IR spectra of [N_2,2,2,2_][TbCl_4_]; Figure S17: FT-IR spectra of [P_4,4,4,4_][TbCl_4_].

## Sample Availability

Samples of the compounds are not available from the authors.
