# Supplementary material for: Lanthanide-Based Organic Salts: Synthesis, Characterization, and Cytotoxicity Studies
Source: Molecules. 2023 Oct 18;28(20):7152. doi: 10.3390/molecules28207152 (PMC10608950; doi:10.3390/molecules28207152)
Supplement: Supplementary file 1 [file molecules-28-07152-s001.zip › molecules-2562810-supplementary.pdf]

Supplementary Materials

Lanthanides-based organic salts: Synthesis, characterization and cytotoxicity studies

Structural characterization of magnetic organic salts (FT-IR)

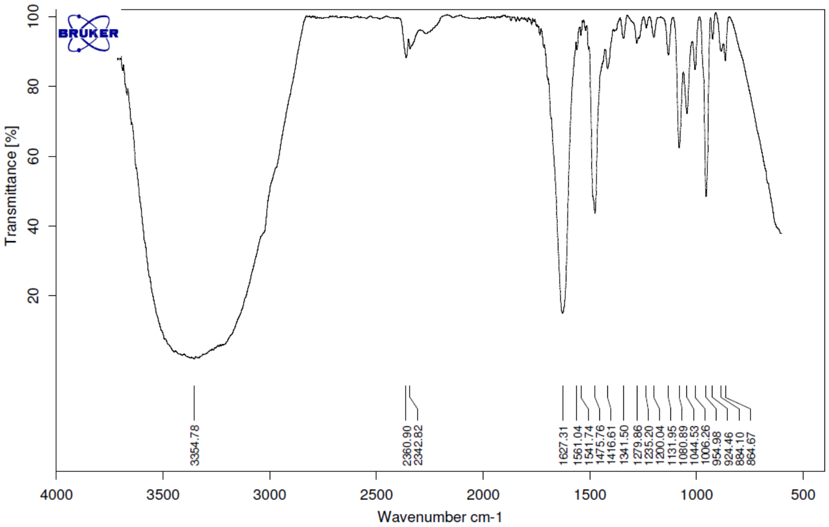

Figure S1. FT-IR spectra of $[N_{1,1,1,C2OH}][GdCl_4]$.

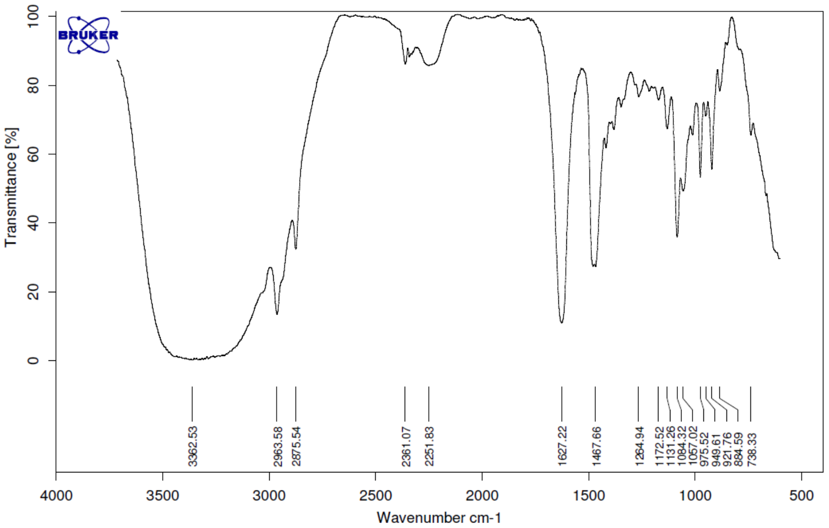

Figure S2. FT-IR spectra of $[N_{1,1,4,C2OH}][GdCl_4]$.

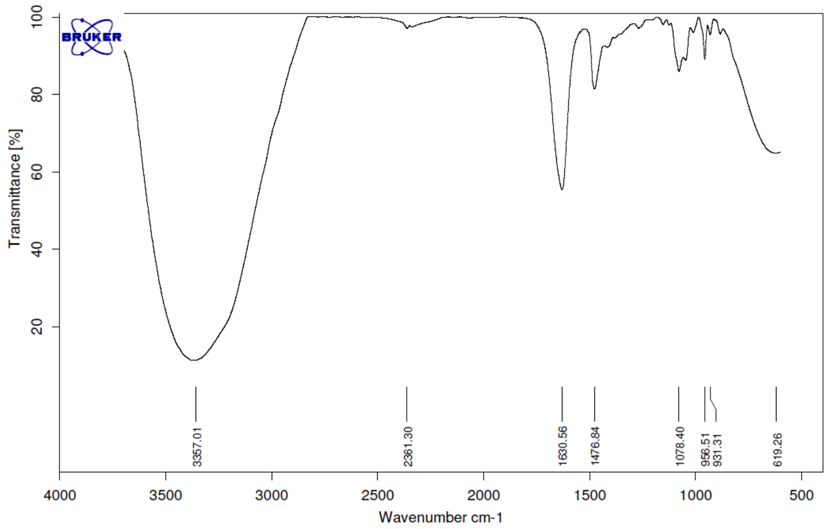

Figure S3. FT-IR spectra of $[N_{1,1,C2OH,C2OH}][GdCl_4]$.

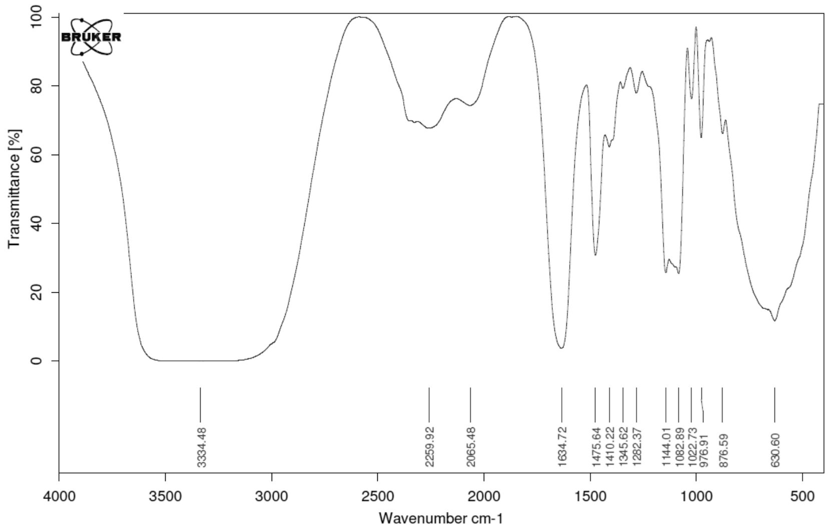

Figure S4. FT-IR spectra of $[N_{1,1,2,C3OH}][GdCl_4]$.

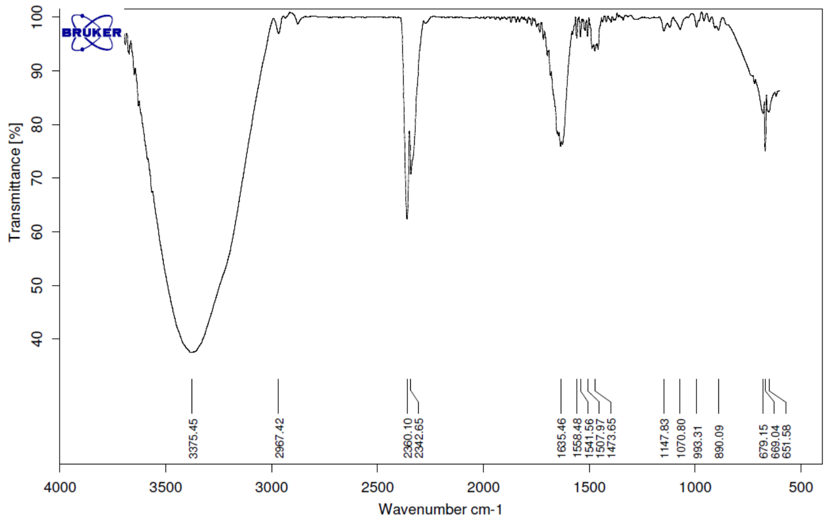

Figure S5. FT-IR spectra of $[N_{1,1,4,C3OH}][GdCl_4]$.

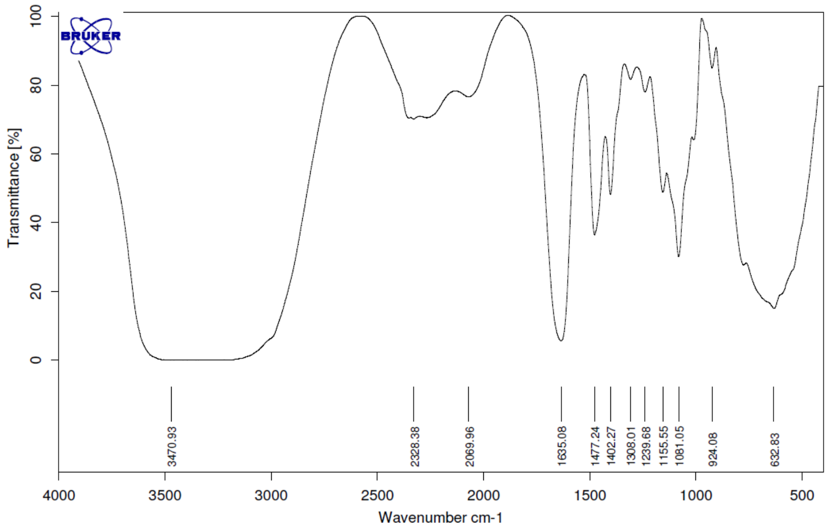

Figure S6. FT-IR spectra of [$N_{2,2,2,C2OH}$][$GdCl_4$].

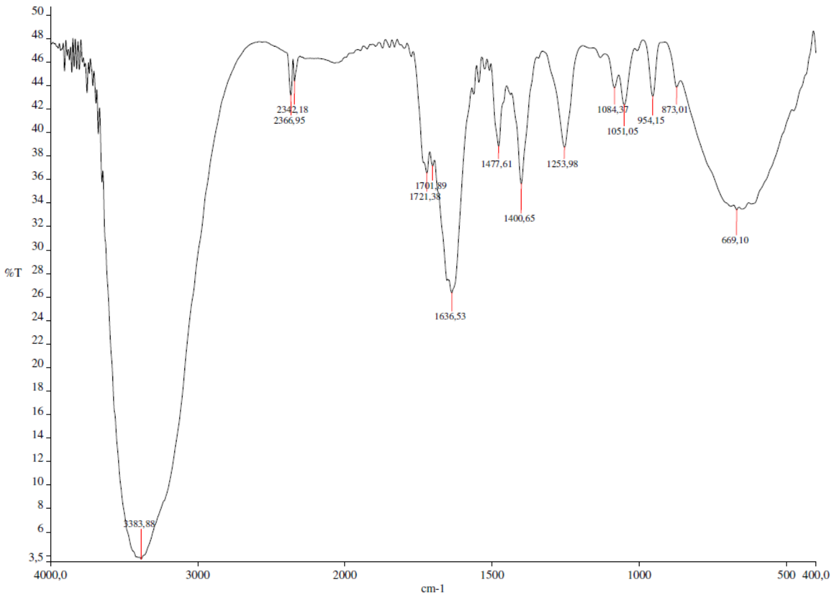

Figure S7. FT-IR spectra of [$N_{1,1,1,C2COOCH3}$][$GdCl_4$].

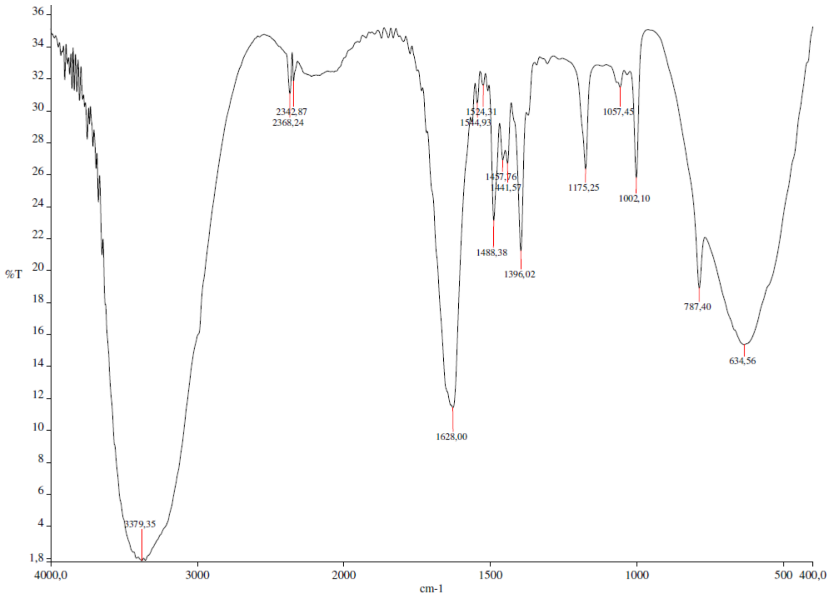

Figure S8. FT-IR spectra of $[N_{2,2,2,2}][GdCl_4]$.

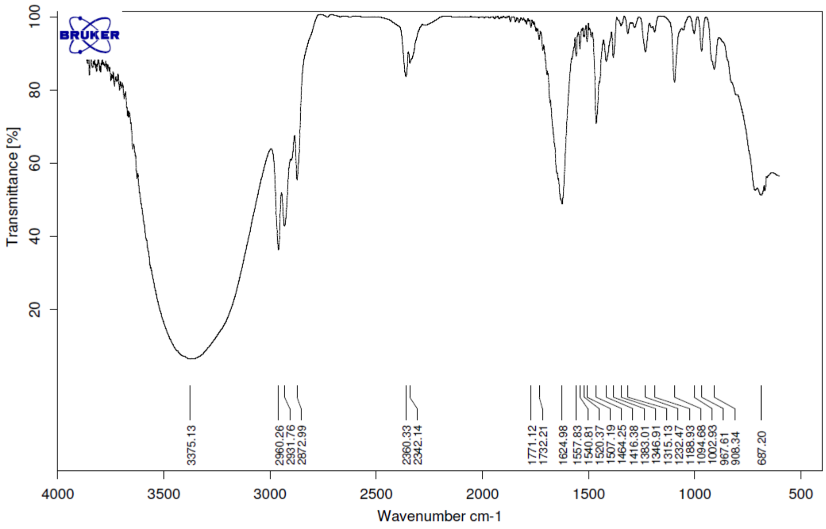

Figure S9. FT-IR spectra of $[P_{4,4,4,4}][GdCl_4]$.

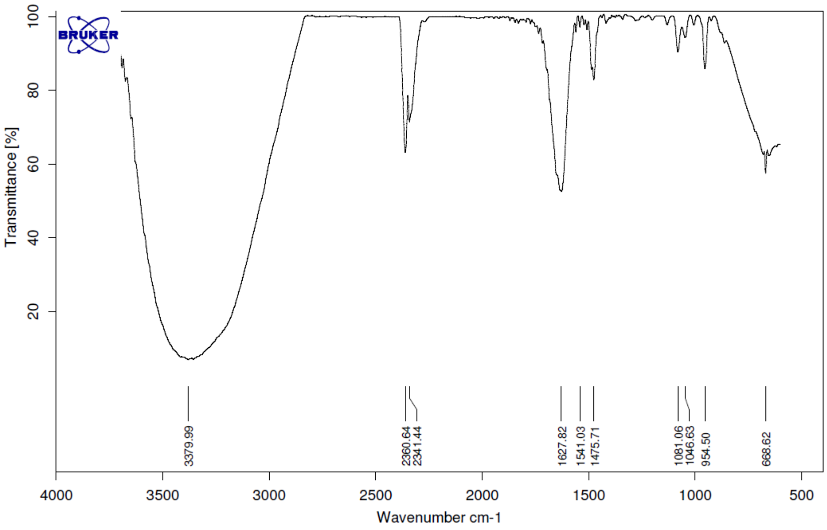

Figure S10. FT-IR spectra of $[N_{1,1,1,C2OH}][TbCl_4]$.

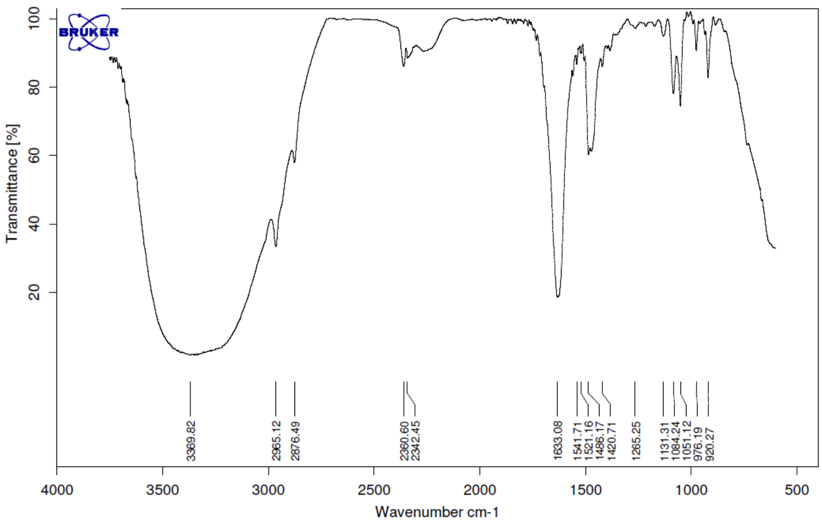

Figure S11. FT-IR spectra of [$N_{1,1,4,C2OH}$][$TbCl_4$].

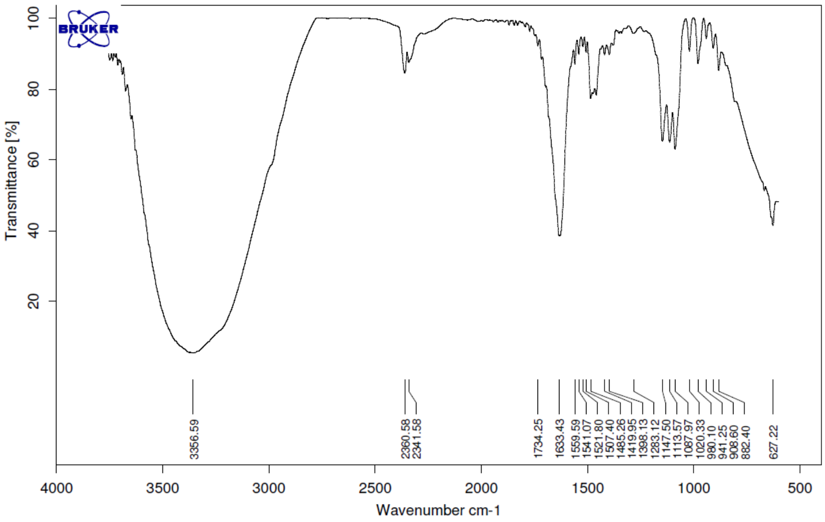

Figure S12. FT-IR spectra of [$N_{1,1,2,C3OH}$][$TbCl_4$].

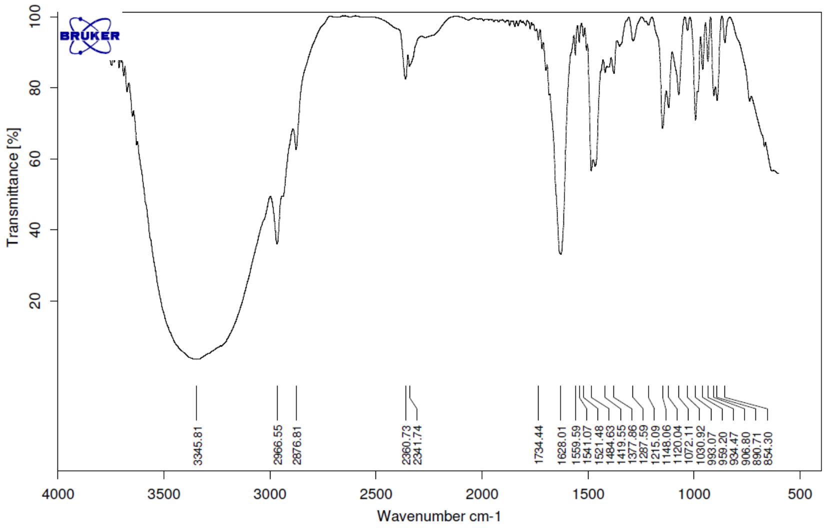

Figure S13. FT-IR spectra of [$N_{1,1,4,C3OH}$][$TbCl_4$].

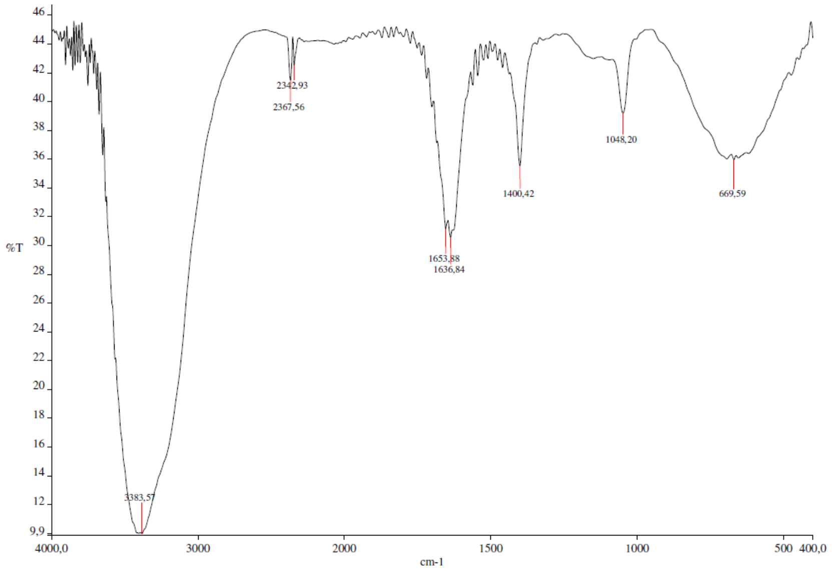

Figure S14. FT-IR spectra of $[P_{C2OH,C2OH,C2OH,C2OH}][TbCl_4]$.

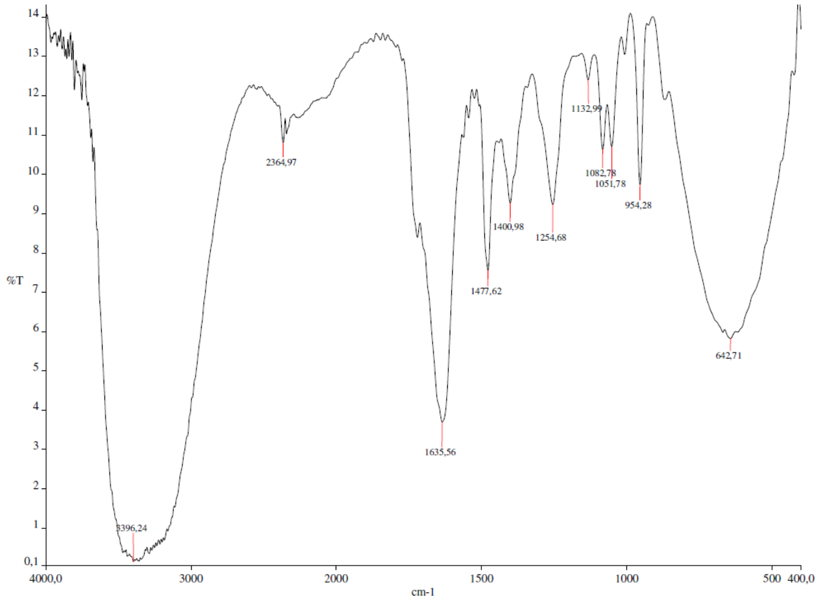

Figure S15. FT-IR spectra of $[N_{1,1,1,C2COOCH3}][TbCl_4]$.

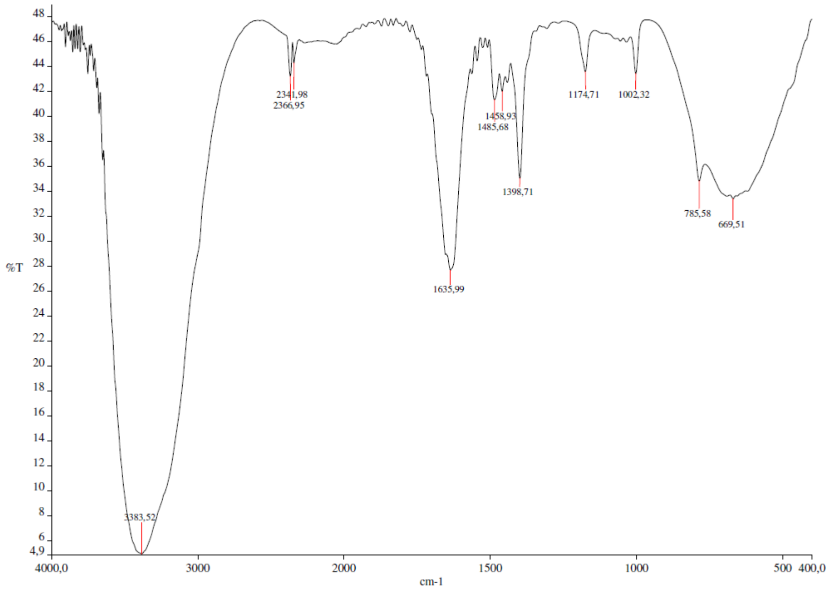

Figure S16. FT-IR spectra of [$N_{2,2,2,2}$][$TbCl_4$].

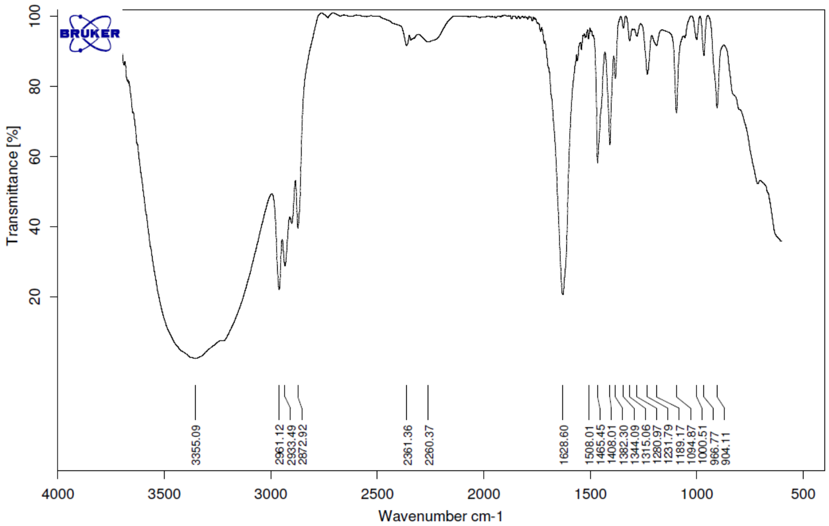

Figure S17. FT-IR spectra of [$P_{4,4,4,4}$][$TbCl_4$].
